# Efficacy of Kinesiotaping on Functional Outcomes, Pain, and Edema in the Early Rehabilitation After Total Knee Arthroplasty Surgery: A Randomized Controlled Trial

**DOI:** 10.3390/jcm13237376

**Published:** 2024-12-04

**Authors:** Francesco Negrini, Edoardo Fascio, Valentina Tivolesi, Catia Pelosi, Elena Tripodo, Giuseppe Banfi, Stefano Negrini, Jacopo A. Vitale

**Affiliations:** 1 Istituti Clinici Scientifici Maugeri IRCCS, 21049 Tradate, Italy; francesco.negrini@uninsubria.it; 2 Department of Biotechnology and Life Sciences, University of Insubria, 21100 Varese, Italy; 3 IRCCS Istituto Ortopedico Galeazzi, 20161 Milan, Italy; edoardo.fascio@gmail.com (E.F.); valentina678.vvt@gmail.com (V.T.); catia.pelosi@grupposandonato.it (C.P.); elena.tripodo@grupposandonato.it (E.T.); banfi.giuseppe@fondazionesanraffaele.it (G.B.); 4 Faculty of Medicine and Surgery, Vita-Salute San Raffaele University, 20132 Milan, Italy; 5 Department of Biomedical, Surgical and Dental Sciences, University “La Statale”, 20122 Milan, Italy; 6 Schulthess Klinik, 8008 Zürich, Switzerland; jacopo.vitale@kws.ch

**Keywords:** rehabilitation, Kinesiotaping, knee surgery, edema, pain

## Abstract

**Background/Objectives**: The aim of our study was to verify whether the application of Kinesiotaping in addition to the usual treatment was superior to the usual treatment alone regarding functional outcome, pain, and edema in the first 13 days after total knee arthroplasty (TKA) surgery. **Methods**: The study sample (*n* = 71) comprised 42.3% men and the mean age was 68.1 (±9) years. A 1:1 ratio randomization list was used to allocate the patient either to a Kinesiotaping Group (KT) or to a control (CON) group. The KT and CON groups received the same volume of standard post-TKA rehabilitation. KT was additionally treated with lymphatic correction applications of Kinesiotaping (Kinesio^®^ Tex Classic, Mogliano Veneto, Italy) on day 3 (±1) and 7 (±1) post-surgery (two applications during the rehabilitation period). Each application lasted four to five days before removal. KT was removed before the patients’ discharge. The main outcome measures were as follows: (1) the circumference at knee level; (2) the VAS for pain; (3) the 10 m Walking Test; (4) the Timed Up and Go Test; (5) the passive knee range of motion; (6) body composition; (7) the Functional Independence Measure; and (8) the Modified Barthel Index. Data were collected at T0 (before surgery), T1 (3 ± 1 days after surgery), T2 (7 ± 1 days after surgery), and T3 (13 ± 1 days after surgery). **Results**: No inter-group differences were found between KT and CON at T0, T1, T2, and T3. An effect of time was observed for all outcome measures. **Conclusions**: No superiority of Kinesiotaping was observed compared to the usual rehabilitation treatment.

## 1. Introduction

According to the World Health Organization, osteoarthritis is the sixth most frequent cause of disability worldwide, due to pain and loss of function [1]. In Italy, osteoarthritis affects the knee in around 60% of subjects older than 65 years old [2]. Surgical treatment is advised in patients suffering from severe knee osteoarthritis, if conservative treatment did not offer sufficient benefit with persisting disability [3]. Total knee arthroplasty (TKA) is the surgical treatment with the highest recommendation for severe osteoarthritis [4,5,6,7,8], with many expected benefits such as pain reduction, and functional and quality of life improvements [9,10,11,12,13]. One of the most common post-surgical complications that can slow down early rehabilitation is post-operative edema. In a recent study by Szöts et al., it was estimated that over 90% of operated patients have post-operative edema [14]. Severe post-operative edema can cause increased pain, decreased ROM, and, ultimately, a worse functional outcome [15]. For the reduction of post-operative edema, many different therapies have been hypothesized: cryotherapy [16], continuous passive mobilization (CPM) [17], compression therapy [15], and pharmacological therapy with selective inhibitors of cyclo-oxygenase 2 [18]. However, none of these therapies have proven to be decisive and there is still room for new conservative therapies aimed at reducing post-operative edema, and speeding up recovery and improving pain, mobility, and independence in post-TKA patients. The quantification of edema in TKA patients is generally performed using clinical measures [19]. However, it has been shown that the segmental impedance measurement of free water can be an objective and effective method for measuring edema after TKA [20].

A technique that can theoretically be effective for reducing edema is Kinesiotaping [21]. It consists of using a particular type of tape, called Kinesio^®^ Tex, and applying it with a specific method. In 2014, a randomized controlled trial (RCT) was published on the efficacy of Kinesiotaping on post-operative edema. According to this study, the technique seemed to be effective in reducing pain and edema, and improving ROM in the post-operative period [19]. A study by Woźniak-Czekierda et al. shows that the use of Kinesiotaping can improve the balance and walking ability in TKA patients [22]. Tornatore et al. conducted in 2020 a three-armed RCT where they compared Kinesiotaping alone vs. lymphatic drainage alone vs. the combined use of Kinesiotaping and lymphatic drainage, showing, in the latter group, higher improvements for pain and leg circumferences six days after surgery [23]. However, to this date, the evidence is still considered inconclusive and not sufficient for advising the regular use of Kinesiotaping in post-TKA patients [24]. Despite the lack of evidence in post-operative edema, a recent meta-analysis highlighted that Kinesiotaping appears to be effective in reducing acute edema of the face and lower limbs in both the short and long term [25]. For this reason, greater clarity is needed regarding the effectiveness of this therapy on edema in the aftermath of TKA. The primary aim of our study was to verify whether the application of Kinesiotaping in addition to usual treatment was superior to the usual treatment alone regarding edema in the first 13 days after TKA surgery, while the secondary aim was to check the efficacy of the aforementioned treatment on functional outcomes and pain. We hypothesized that we would find reduced edema 13 days after surgery in patients treated with Kinesiotaping.

## 2. Materials and Methods

### 2.1. Study Design

An open-label, parallel, two-group, randomized controlled trial was conducted between May 2018 and February 2020 at IRCCS Istituto Ortopedico Galeazzi hospital. The study was approved by the Ethics Committee of Vita-Salute San Raffaele University (Ref. 61/int/2018) and all study procedures were performed in accordance with the Declaration of Helsinki. All subjects received clear explanation of purpose, methods, potential risks, and benefits of the study, and, before the beginning of the experimental procedures, written informed consent was obtained from all the participants. The study protocol was previously registered on ClinicalTrials.gov (NCT03681106) and conducted in accordance with the CONSORT guidelines [26].

### 2.2. Sample Size

At first, a difference of 0.7 cm was considered clinically significant in the operated knee circumference (OKC) between the two groups (i.e., Kinesiotaping vs. control) at the 13th post-operative day. The value of 0.7 cm was selected in line with the data observed in a previous study by Donec et al. [19]. Therefore, assuming a common standard deviation (SD) of 1 cm, a 5% type I error, and a 10% type II error, while taking into account a 10% dropout rate from the whole sample, a total sample size of 98 subjects was planned, with 49 subjects for each group. However, due to the health crisis triggered by the COVID-19 pandemic and the consequent sanitary–organizational arrangements set up for its contrast, it was necessary to interrupt the study prematurely, with a sample size of 71 (experimental = 33; and control = 38), in accordance with the local ethical committee. A post hoc power analysis on the completed sample, conducted to account for the impact of the COVID-19 pandemic, certifies a statistical power of 83% (α = 0.05, and d = 0.7).

### 2.3. Participants

**Figure 1:**
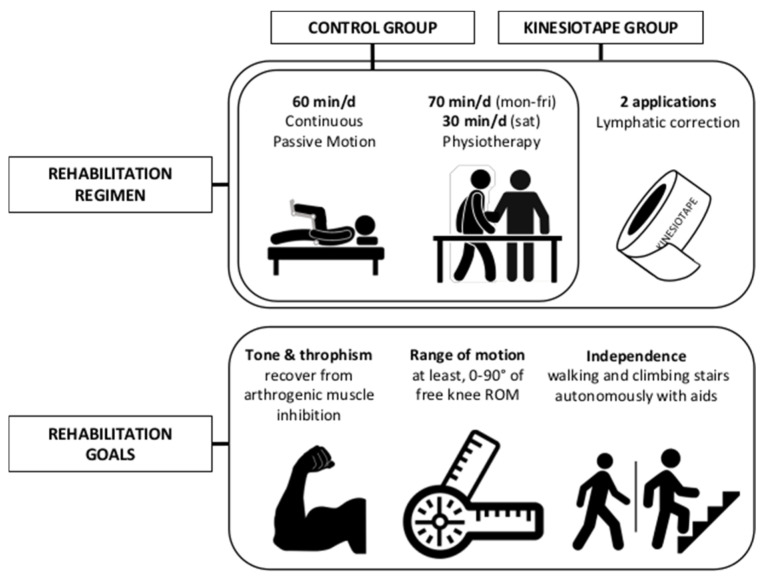
Standard rehabilitation regimen following TKA plus experimental intervention. min/d = minutes/day.

Inpatients, aged between 50 and 85 years old, waiting for total knee arthroplasty (TKA) and scheduled to receive TKA and rehabilitation, were invited by the principal investigator (FN) to voluntarily participate to the trial. Recruitment proceeded following the inpatients’ order of presentation and their acceptance of the written informed consent, until the sample size was reached. Patients were recruited in the rehabilitation ward of a specialized orthopedic hospital. A 1:1 ratio randomization list (simple randomization), to allocate the patient either to a Kinesiotaping Group (KT) or to a control (CON) group, was entrusted to an independent investigator (JV). The randomization list was produced using a web-based system and was concealed from all the investigators included in the recruitment, assessment, and treatment delivery. The exclusion criteria were as follows: Barthel Index < 70, BMI > 35, stage 3 and 4 congestive heart failure, stage 3 and 4 chronic kidney disease, fragile skin, active skin infection or open skin lesion at the application site, sensitivity or allergy to the tape, pregnancy, and revision surgery. During a fortnight’s hospitalization period after the surgery, both KT and CON groups received the same amount and methods of rehabilitation administered by the same rehabilitation team. Patients in both groups underwent 60 min per day of Continuous Passive Motion therapy, along with 70 min of physiotherapy from Monday to Friday, and 30 min on Saturday. The treatment included strengthening and mobilization exercises of the affected limb, exercise to improve global motor control, and functional exercise such as walking and climbing stairs. KT group was additionally treated with lymphatic correction applications of Kinesiotaping (Kinesio^®^ Tex Classic, Mogliano Veneto, Italy) on day 3 (±1) and 7 (±1) post-surgery (2 applications during the rehabilitation period). Figure 1 summarizes rehabilitation regimens.

### 2.4. Lymphatic Correction

**Figure 2:**
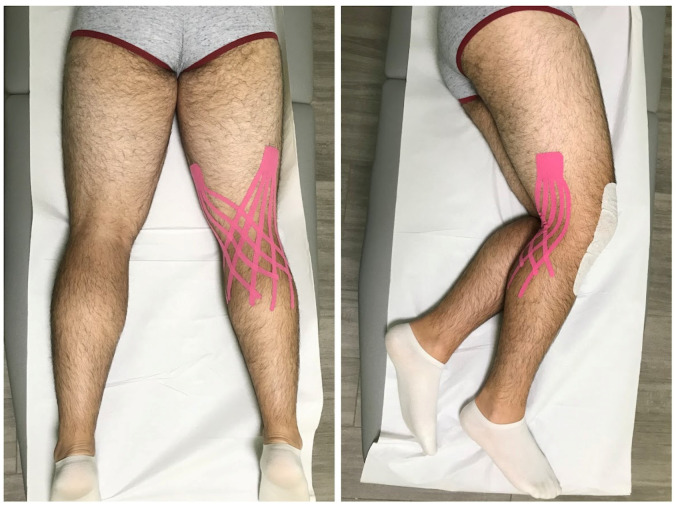
Posterior and lateral view of KT application in the intervention group.

Lymphatic correction was performed by two skilled KT practitioners (VT and EF). Two Fan strips with five tails were applied to the posterior aspect of the fully extended operated limb. One originated above the lateral condyle of the femur and the other above the medial one. Then, both crossed over the popliteal space and cross each other, forming a criss-cross pattern, ending over the region of swelling (Figure 2). Neither the base and the tips nor the stripe were tensioned [27]. Each application lasted 4–5 days before removal. KT was removed before patients’ discharge.

### 2.5. Outcomes

The primary outcome of the study was the circumference at the level of the knee of the operated limb (OKC) thirteen days after surgery. Secondary outcomes were pain (Visual Analogue Scale for pain (VAS) 0–10 cm) [28], functional performance (10 m Walking Test [29], and Timed Up and Go Test [30]), knee range of motion (passive range of motion measured with manual goniometer), body composition (total and segmental bioimpedance analysis) [20], and functional independence (Functional Independence Measure (FIM) 0–126 [31], and Modified Barthel Index 0–100 [32]).

### 2.6. Procedures/Clinical Assessments

**Figure 3:**
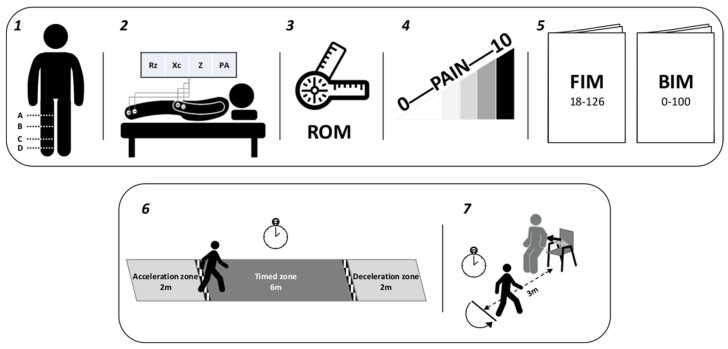
Primary and secondary study outcomes: (1) operated limb circumferences: [A] 10 cm above the superior edge of the patella; [B] at the level of the middle line of articular space; [C] 25 cm above the inferior pole of the lateral malleolus; and [D] 2 cm above the medial malleolus; (2) BIA: Rz = electric resistance, Xc = capacitive reactance, Z = impedance, and PA = phase angle; (3) passive ROM of the operated knee (°); (4) VAS = Visual Analogue Scale; (5) FIM = Functional Independence Measure and BIM: Modified Barthel Index; (6) 10 m Walking Test; and (7) Timed Up and Go Test.

All the aforementioned variables were collected at T0 (before surgery), T1 (3 ± 1 days after surgery), T2 (7 ± 1 days after surgery), and T3 (13 ± 1 days after surgery). All measurements were performed with patients wearing anti-embolism stockings, as part of the post-surgery prescription. Most of the procedures and clinical tools are described elsewhere; Figure 3 summarizes the assessment procedures. A plastic tape measure was used for measurements of the circumference of the operated limb. Passive range of motion (pROM) of the knee was assessed by physiotherapists. The BIA 101-ASE vector impedance analyzer (Akern S.r.l, Pontassieve, Italy) was used; participants were asked to lie down in bed for at least 10 min before the body composition assessments and to remain still during the examination. Evaluators were blinded from group allocation (socks covered the tape) and from the objective of the study. Blinding for participants and providers was not feasible because the tape was visible during the experimental period.

### 2.7. Statistical Analysis

Data analysis was performed using SPSS statistic software (IBM^®^ SPSS^®^ Statistics 20) and interpreted at two-tailed significance level < 0.05. Demographic characteristics are reported as absolute and relative frequencies for categorical variables, as mean with SD for continuous variables. Normality of outcome measures was assessed at each time point and for the two study groups separately with the Shapiro–Wilk test; in accordance, parametric (*t* test) or non-parametric (Mann–Whitney *U* test) methods were applied to compare groups at baseline. Chi-square test was used to assess significant associations between categorical variables. Cohen’s d was used to estimate the effect size of difference between groups at baseline. Analysis of variance (ANOVA) was used to test whether there was an interaction between time (within-group factor) and type of intervention (between-group factor); furthermore, Tukey’s multiple comparison was used. Bonferroni correction was applied. Both ‘per protocol’ and ‘intention to treat’ analysis (using multiple imputation to impute values for the missing data [33]) were performed.

## 3. Results

### 3.1. Participants’ Characteristics

**Figure 4:**
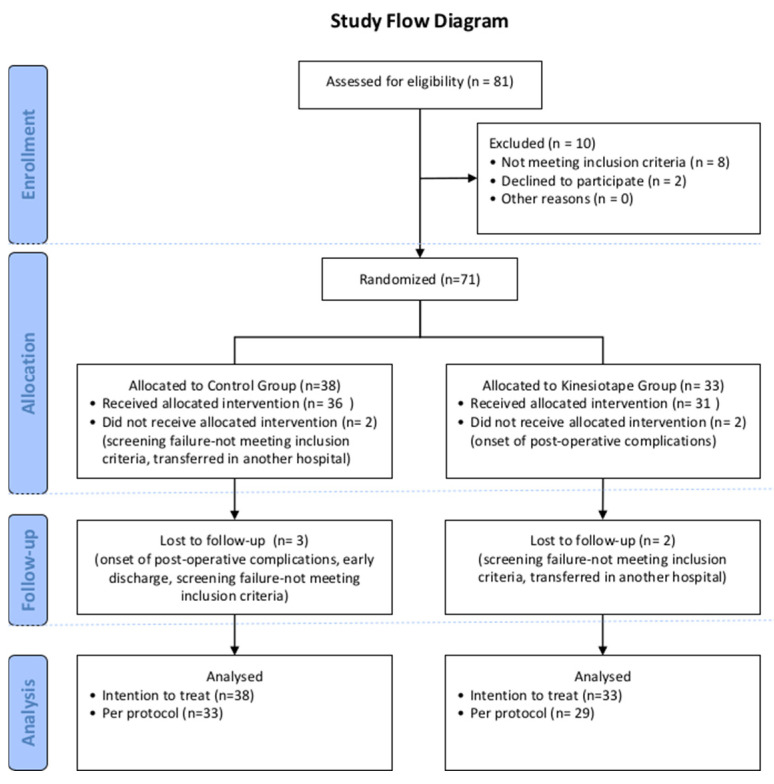
CONSORT study flow diagram.

Figure 4 shows the participants’ flow through the trial. The initial study sample comprised 42.3% men and the mean age was 68.1 (±9) years. Overall, 90% of the values of the variables of interest were collected from 71 participants, whereas 10% of the missing data was imputed by means of the multiple imputation analysis method [33]. Multiple imputation creates multiple complete datasets by filling in missing values based on the observed data patterns, then combining the results to provide robust estimates, making it an effective approach when the missing data are moderate (such as 10%) rather than excessive. At baseline, all the anthropometrical, functional, and clinical variables were similar for both groups (Table 1).

### 3.2. Outcomes Assessment

**Figure 5:**
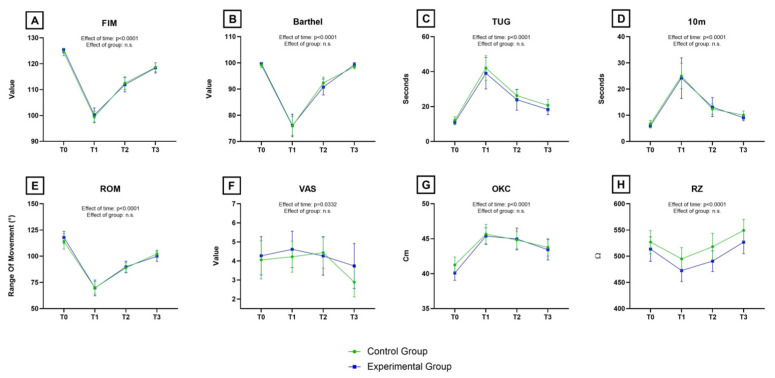
Variation of the means, with error bars representing the 95% CI, of the clinical variables at different time points (T0, T1, T2 and T3). (**A**) FIM = Functional Independence Measure; (**B**) Barthel Index; (**C**) TUG = Timed Up and Go Test; (**D**) 10 m = 10 m walking test; (**E**) ROM = passive flexion of the knee of the operated limb; (**F**) VAS = Visual Analogue Scale for the evaluation of pain in the operated knee; (**G**) OKC = circumference of the knee of the operated limb; and (**H**) RZ = electric resistance. Details on statistical differences for the effect of time are reported in Table 2. n.s. = not significant.

No significant difference was found in OKC between the KT and CON groups thirteen days after surgery (*p* = 0.99, Tukey’s test). No differences in the secondary outcomes were found between the two groups at T3, nor at T0, T1, and T2. The ANOVA showed no significant effect (also after Bonferroni correction) of the type of intervention (group) but a significant effect of time on all the variables (see Table 2 for details). The multi-paneled Figure 5 shows variables’ means with a 95% CI at all different time points. No differences were found between the ‘per protocol’ and ‘intention to treat’ analysis.

### 3.3. Adverse Events or Unintended Effects

No adverse events or unintended effects were observed.

## 4. Discussion

The present study is a large RCT tailored for investigating the efficacy of Kinesiotaping in reducing edema and improving functionality in a very early stage of TKA rehabilitation. We did not find any statistically significant difference between the KT and CON group. Our initial hypotheses were not confirmed. The results of our study differ from the findings of the 2014 RCT by Donec et al. [19] where the KT technique appeared to be beneficial for reducing post-operative pain and edema, and improving knee extension in the early post-operative rehabilitation period. One critical factor contributing to these discrepancies may be the difference in application protocols. In our study, we utilized a criss-cross lymphatic correction method applied in the popliteal fossa, designed specifically to facilitate fluid drainage and reduce edema. Conversely, in the study by Donec et al., the taping technique emphasized a broader application across the anterior knee region, potentially targeting both edema reduction and biomechanical alignment. Another notable difference is the follow-up period. Donec et al. assessed outcomes at 28 days post-operatively, whereas our study focused on the very early post-operative period (13 days). Swelling and pain may respond differently to therapeutic interventions over longer periods [34], particularly as the inflammatory and healing processes evolve. The shorter follow-up in our study may have limited our ability to capture the potential late-stage benefits of Kinesiotaping. These differences suggest that the timing and method of application are critical variables that warrant further investigation to optimize Kinesiotaping for post-operative care. Finally, the patient characteristics and surgical context might also contribute to variability. For example, our study was conducted in a high-volume orthopedic center with standardized rehabilitation protocols, whereas the differences in care delivery settings in other studies could influence outcomes. These factors underscore the importance of contextualizing the results within specific clinical environments.

Notably, out of 71 patients, we did not find a single adverse effect. Kinesiotaping was generally well-tolerated, and no patient had to interrupt the treatment because of dermatological problems. This finding is similar to most of the studies that can be found in the literature [19,22,23]. This expected finding, combined with the low cost of the treatment (a single meter of about 5 cm-width tape costs approximately 5 €), and the mixed results on KT efficacy, gives the scientific community more ethical grounds to research and deepen our understanding of this specific treatment. This variability highlights the importance of developing standardized application protocols for Kinesiotaping.

The RCT by Woźniak-Czekierda et al. [22] used dynamic taping, and the study focused on examining the sensorimotor efficiency, balance, and gait in patients, while our study focused more on reducing edema. The different finality reflects in a different application of the Kinesiotaping. The problem of the correct application of the tape in a technique such as Kinesiotaping is of crucial importance. Because there are different possibilities in applying the tape, there still is no standardization. The study by Woźniak-Czekierda et al. was started 4 to 5 weeks after surgery, when edema is a less prominent complication and the rehabilitative focus is on functional improvement and fall prevention. Furthermore, after 4 weeks, the surgical wound is usually healed and there is no need for medication. The differences in aim and skin availability affect the very different applications of the tape; we applied the tape mainly on the popliteal fossa with a criss-cross pattern in order to focus on the lymphatic drainage of the shank by directing fluid towards the ventromedial and dorsolateral lymphatic bundles, to obtain swelling reduction. On the other hand, in the study by Woźniak-Czekierda [22], the tape was positioned on the more common anterior part of the knee, in order to optimize the knee position and improve the alignment of the biomechanical axis. Because of the important differences in timepoints, and in the way Kinesiotaping was applied, it may be a stretch to compare the results of the two studies.

The discrepancies with the results of previous studies, such as that by Donec et al. (2014) [19], may be due to hte differences in application protocols or follow-up periods. Studies like that of Woźniak-Czekierda et al. (2017) [22], where Kinesiotaping was used to improve balance and gait, suggest that the effectiveness of the treatment may vary depending on the goal and timing of rehabilitation. This variability highlights the importance of developing standardized application protocols for Kinesiotaping.

A possible key to correctly understand the results of the study can be found in the three-way RCT by Tornatore et al. [23]. Their RCT analyzed the effect of Kinesiotaping and lymphatic drainage, both alone and combined, in an early phase of rehabilitation (up to 6 days after surgery). They found a consistent advantage in combining the two treatments, as they appear to be synergic. It is possible to hypothesize that combining the two methods can give benefits even after 13 days. Unfortunately, our study did not find an advantage in applying Kinesiotaping in an early phase after TKA.

Although our findings do not support routine use of Kinesiotaping in the early post-operative period after TKA, the intervention was well-tolerated and associated with no adverse effects, making it a low-risk option for exploration in other contexts. For example, future studies could investigate Kinesiotaping in combination with other therapies, such as manual lymphatic drainage or advanced physical therapy techniques, to explore synergistic effects. Additionally, applying Kinesiotaping during the later stages of rehabilitation, when functional restoration and biomechanical alignment are the primary goals, may yield different outcomes. Hypothetically, Kinesiotaping could also be adapted for use in patients undergoing other types of lower-limb orthopedic procedures, such as hip replacements or ligament reconstructions, where edema and pain management are critical. Moreover, advancements in tape technology or application methods might enhance its therapeutic potential. For instance, integrating Kinesiotaping with wearable sensors to monitor limb swelling or mobility could provide real-time feedback and enable more personalized rehabilitation strategies.

The study we conducted has many strengths, the first of which is the study design and methodology. This is indeed an RCT with a considerable number of patients involved, which allowed us to form homogenous groups both in demographics and clinical situation. We carefully selected only patients undergoing TKA for the first time for osteoarthritis. We conducted our study in an important Italian orthopedic hospital that counts almost four thousand joint arthroplasties each year; for this reason, the rehabilitative team involved had considerable experience in TKA rehabilitation. Kinesiotaping was applied by trained physiotherapists in a standardized and repeatable manner and with a precise and predetermined timeline. Furthermore, we investigated the crucial early phase after surgery.

### Study Limitations

We need to recognize a few limitations. Due to the COVID-19 pandemic, which severely impacted clinical practice, we fell just short of the expected sample size, including 71 patients instead of 98. A reduced sample size, although justified by a power analysis, amplifies the possibility of a type II error, which could partly explain the lack of a statistically significant difference in outcome between the two groups. On the other hand, our post hoc analysis certifies a statistical power of 83% (α = 0.05, d = 0.7), enough to provide a strong clue that our results were not due to chance. Furthermore, the lack of a longer follow-up can be considered a limitation. Lastly, blinding participants and providers was not feasible in this study due to the visible nature of the Kinesiotaping intervention. While evaluators were blinded to group allocation during the outcome assessments (e.g., tape concealed under anti-embolism stockings), the lack of participant and provider blinding may have introduced performance and detection biases. Specifically, participants in the intervention group may have experienced placebo effects, potentially influencing subjective measures such as pain scores. Similarly, providers aware of group allocation might have unconsciously modified their delivery of standard rehabilitation treatments, impacting the functional outcomes. These biases, though unintended, are inherent challenges in studies of this nature. To mitigate these biases in future studies, alternative strategies could be considered. For example, incorporating a sham intervention group using placebo taping (e.g., taping with no therapeutic purpose) may help control for placebo effects. Additionally, increasing the standardization of provider-delivered care through structured protocols or supervision by blinded supervisors may reduce potential performance bias. Such approaches could strengthen the validity of future research findings.

Generalizability: Our study sample was composed primarily of patients undergoing primary TKA at a specialized orthopedic hospital. This setting ensures that participants received consistent, high-quality surgical care and rehabilitation. However, this level of standardization may limit the applicability of our findings to broader populations, particularly those in non-specialized or resource-limited settings. For instance, patients undergoing TKA in community hospitals or clinics with a less robust rehabilitation infrastructure might experience different outcomes with or without Kinesiotaping. Additionally, the exclusion of patients with significant comorbidities, such as advanced heart failure or chronic kidney disease, further narrows the scope of generalizability. These comorbidities are common in older adults, who represent a significant proportion of the TKA population. Thus, the impact of Kinesiotaping on patients with more complex health profiles remains uncertain and warrants further investigation. It is also noteworthy that our study focused on the early post-operative period, and findings may not directly translate to later stages of rehabilitation or other clinical conditions where Kinesiotaping might be applied differently. For example, its effectiveness in managing chronic post-operative swelling or improving biomechanical alignment in patients with recurrent or revision surgeries has not been addressed. Finally, cultural and healthcare system differences could influence the feasibility and adoption of Kinesiotaping. For example, in healthcare systems where patient access to physiotherapy is limited, a low-cost, self-administered intervention like Kinesiotaping might offer additional benefits when used in conjunction with home-based exercise regimens. Conversely, in systems with abundant physiotherapy resources, the relative benefit of Kinesiotaping might be less pronounced. Future studies should aim to replicate our findings in diverse clinical settings, including community hospitals, outpatient clinics, and populations with varied healthcare access. Additionally, exploring the role of Kinesiotaping in different cultural contexts and healthcare models could provide a broader understanding of its potential applications and limitations.

## 5. Conclusions

Because of the results of our study, we cannot recommend the use of Kinesiotaping applied in the popliteal fossa in the early stages of rehabilitation after TKA. However, given the low cost and safety of Kinesiotaping, it should be important to investigate the possible role of Kinesiotaping both in the successive phases of rehabilitation, or associated with other kinds of orthopedic surgery, and in combination with other treatments, such as physical or manual therapies.

## Figures and Tables

**Table 1:** Patients’ characteristics at baseline.

	CON (*n* = 38)	KT (*n* = 33)	*p*-Value *	Cohen’s d	Category
Men—*n* (%)	15 (39.5%)	15 (45.5%)	0.638	//	//
Right knee TKA—*n* (%)	23 (60.53%)	15 (45.45%)	0.239	//	//
Age (years)	68.6 ± 10.1	67.5 ± 7.5	0.593	0.122	Trivial
BMI (Kg/h^2^)	28.15 ± 3.87	27.82 ± 3.1	0.693	0.093	Trivial
RZ (Ω)	527 ± 63	513 ± 61	0.810	0.226	Small
OKC (cm)	41.2 ± 3.2	40.1 ± 2.8	0.489	0.364	Small
VAS (0–10)	4.1 ± 2.8	4.2 ± 2.7	0.426	−0.036	Trivial
TUG (s)	12.1 ± 5.6	10.9 ± 3.4	0.748	0.255	Small
10 m (s)	6.7 ± 3.6	5.4 ± 1.8	0.776	0.447	Small
MBI (0–100)	99.1 ± 2.9	99.7 ± 1.1	0.854	−0.266	Small
FIM (18–126)	126 ± 4	126 ±1	0.454		

Plus–minus values are the mean ± standard deviation. TKA = total knee arthroplasty, BMI = Body Mass Index, RZ = electric resistance, OKC = circumference of the knee of the operated limb, VAS = Visual Analogue Scale for pain, TUG = Timed Up and Go Test, 10 m = 10 m Walking Test, MBI = Modified Barthel Index, FIM = Functional Independence Measure. * Chi-square test or *t* test, as appropriate.

**Table 2:** Between and within-group comparisons (KT vs. CON) at different time points (T0, T1, T2, and T3). Between parentheses, 95% Confidence Interval. * = significant values.

	KT				CON							
Variables	T0	T1	T2	T3	T0	T1	T2	T3	Effect of Time	Contrast	Effect of Group	Interaction
FIM (18–126)	125.5 ± 1.0 (125.1, 125.9)	100.2 ± 6.8	111.9 ± 7.1	118.4 ± 5.1 (116.4, 120.3)	124.5 ± 3.9 (123.1, 125.9)	99.4 ± 6.2	112.5 ± 6.7	118.6 ± 4.8 (116.9, 120.37)	<0.001 *	-	n.s.	n.s.
MBI (0–100)	99.7 ± 1.0 (99.3, 100.1)	76.1 ± 11.1	90.8 ± 8.0	99.3 ± 2.2 (98.4, 100.1)	99.1 ± 2.9 (98.1, 100.2)	75.9 ± 9.8	92.3 ± 6.1	98.5 ± 2.2 (97.7, 99.3)	<0.001 *	CON: T0 = T3 (*p* > 0.05) ^a^ KT: T0 = T3 (*p* > 0.05) ^a^	n.s.	n.s.
TUG (s)	10.9 ± 3.4 (9.6, 12.2)	39.0 ± 20.8	23.9 ± 15.4	18.3 ± 7.6 (15.4, 21.2)	12.1 ± 5.6 (10.1, 14.1)	42.0 ± 18.6	26.2 ± 9.4	20.6 ± 9.4 (17.2, 24.0)	<0.001 *	-	n.s.	n.s.
10 m (s)	5.3 ± 1.8 (4.7, 6.0)	24.1 ± 18.3	13.1 ± 9.4	9.0 ± 3.0 (7.9, 10.2)	6.7 ± 3.6 (5.4, 8.0)	25.0 ± 13.2	12.4 ± 5.3	10.0 ± 4.5 (8.4, 11.6)	<0.001 *	-	n.s.	n.s.
ROM (°)	117.9 ± 15.1 (112.2, 123.7)	69.7 ± 19.2	89.9 ± 13.6	3.7 ± 3.0 (99.9, 106.8)	114.0 ± 20.5 (106.8, 121.4)	69.8 ± 16.4	88.9 ± 13.5	102.2 ± 9.7 (98.7, 105.7)	<0.001 *	-	n.s.	n.s.
VAS (0–10)	4.2 ± 2.6 (3.2, 5.2)	4.6 ± 2.4	4.2 ± 2.6	3.7 ± 3.0 (2.5, 4.9)	4.0 ± 2.8 (3.0, 5.0)	4.2 ± 2.3	4.4 ± 2.2	2.9 ± 2.1 (2.1, 3.6)	0.033 *	CON: T0 = T1 = T2; T0 = T3 (*p* > 0.05) ^a^ KT: T0 = T1 = T2 = T3 (*p* > 0.05) ^a^	n.s.	n.s.
OKC (cm)	40.1 ± 2.7 (39.0, 41.1)	45.3 ± 3.0	44.9 ± 4.0	43.4 ± 3.7 (41.9, 44.8)	41.2 ± 3.2 (40.1, 42.4)	45.6 ± 3.8	44.8 ± 3.6	42.7 ± 3.4 (41.5, 44.0)	<0.001 *	CON: T1 = T2; T2 = T3 (*p* > 0.05) ^a^ KT: T1 = T2 (*p* > 0.05) ^a^	n.s.	n.s.
RZ (Ω)	513.5 ± 61.0 (490.3, 536.7)	472.6 ± 54.1	490.6 ± 49.7	527.0 ± 57.2 (505.2, 548.7)	527.0 ± 62.5 (504.7, 549.2)	494.9 ± 60.2	518.1 ± 71.9	549.3 ± 58.5 (528.5, 570.2)	<0.001 *	CON: T0 = T2; T0 = T3 (*p* > 0.05) ^a^ KT: T0 = T2; T0 = T3; T1 = T2 (*p* > 0.05) ^a^	n.s.	n.s.

Data are reported as mean ± SD. ^a^: Tukey’s post hoc test, only not significant comparison are shown; n.s.: non-significant; CON: Control Group; KT: Experimental Group. FIM = Functional Independence Measure; MBI = Modified Barthel Index; TUG = Timed Up and Go Test; 10 m = 10 m Walking Test; ROM: passive knee Range Of Motion; VAS = Visual Analogue Scale for pain; OKC = circumference of the knee of the operated limb; and RZ = electric resistance.

## Data Availability

Data are available in a public, open-access repository: https://zenodo.org/record/7655928#.Y_MoWezMIdU, access on 1 November 2024.
